# Argatroban in the management of heparin-induced thrombocytopenia: a multicenter clinical trial

**DOI:** 10.1186/s13054-015-1109-0

**Published:** 2015-11-11

**Authors:** Brigitte Tardy-Poncet, Philippe Nguyen, Jean-Claude Thiranos, Pierre-Emmanuel Morange, Christine Biron-Andréani, Yves Gruel, Jérome Morel, Alain Wynckel, Lelia Grunebaum, Judith Villacorta-Torres, Sandrine Grosjean, Emmanuel de Maistre

**Affiliations:** EA 3065, Université Jean-Monnet, INSERM CIC 1408 - FCRIN-INNOVTE - Laboratory of Hematology, University Hospital of St Etienne, St Etienne, France; Laboratory of Hematology, University Hospital of Reims, Reims, France; Department of Cardiovascular Surgery, University Hospitals of Strasbourg, Strasbourg, France; Laboratory of Hematology, University Hospital of Marseille, Marseille, France; Laboratory of Hematology, University Hospital of Montpellier, Montpellier, France; Laboratory of Hematology, University Hospital of Tours, Tours, France; Department of Intensive Care Unit, University Hospital of St Etienne, St Etienne, France; University Hospital of Reims, Reims, France; Laboratory of Hematology, University Hospitals of Strasbourg, Strasbourg, France; Intensive Care Unit, University Hospital of Marseille, Marseille, France; Intensive Care Medicine, University Hospital of Dijon, Dijon, France; Laboratory of Hematology, University Hospital of Dijon, Dijon, France

## Abstract

**Introduction:**

The aim of this study was to collect data in France in patients with heparin-induced thrombocytopenia who required parenteral anticoagulation and for whom other non-heparin anticoagulant therapies were contraindicated including patients with renal failure, cross-reactivity to danaparoid or at high hemorrhagic risk.

**Methods:**

A total of 20 patients, of mean age 72 ± 10 years, were enrolled in this open-label, multicenter clinical study. Exploratory statistical data analysis was performed with descriptive interpretation of intra-individual comparisons using simple univariate statistics.

**Results:**

The diagnosis of HIT was confirmed in 16 subjects by an independent scientific committee. Fourteen patients (70 %) were in an intensive care unit during the course of the study. Patients were treated with argatroban for a mean duration of 8.5 ± 6.1 days. The mean starting dose of argatroban was 0.77 ± 0.45 μg/kg/min. Platelet recovery was rapid.

aPTT and anti-IIa activity assays were used to monitor the dose of argatroban. The mean baseline aPTT value was 45.0 ± 9.8 sec and increased to 78.2 ± 35.8 sec two hours after initiating argatroban. At this time mean argatroban concentration was 0.34 ± 0.16 and 0.61 ± 0.28 μg/ml using ECT and TT measurements, respectively. New and/or extended thromboses were reported in 25 % of patients and major bleedings were documented in 15 %. Six patients died due to their underlying medical condition.

**Conclusion:**

Considering its hepatic elimination and its short half-life, argatroban can be considered as a safe therapeutic option in HIT patients at high hemorrhagic risk and with renal failure, particularly in an ICU setting.

## Introduction

Heparin is one of the most widely used anticoagulants for a variety of clinical conditions. One serious complication of heparin therapy is immune-mediated heparin-induced thrombocytopenia (HIT), which can be associated with subsequent venous and arterial thrombosis. For HIT patients an alternative parenteral anticoagulant is always needed for the prevention of the thrombotic risk associated with HIT and for the treatment of thrombosis related to HIT, or for continued anticoagulation. In France the current authorized treatment options for patients with HIT are danaparoid and argatroban. Danaparoid is a heparinoid factor Xa inhibitor [1]. It is renally eliminated and has a long elimination half-life, increasing the bleeding risk [2]. In addition, cross-reactivity between danaparoid and heparin antibodies can occur and subsequently lead to new complications and unsatisfactory outcomes [3].

Argatroban is a synthetic thrombin inhibitor that offers potential benefit to patients with HIT. Argatroban is unique among direct thrombin inhibitors (and other anticoagulants) because it is predominantly hepatically metabolized, acts rapidly and has a short elimination half-life of 52 ± 16 minutes, ensuring rapid restoration of hemostasis upon cessation of treatment [4], which is of particular interest in elderly patients like those in our study. Argatroban does not cross-react with heparin-dependent antibodies and does not induce antibody formation [5].

Recently, the American College of Chest Physicians Clinical Practice Guidelines [6] and Nordic Expert Panel Clinical and Laboratory Guidelines [7] recommended the use of argatroban in treating HIT patients with thrombosis and renal failure rather than other non-heparin anticoagulants.

Argatroban was approved in Europe in 2004 [8]. The aim of this study was to collect data in France in patients with HIT who required parenteral anticoagulation and for whom other non-heparin anticoagulant therapies were contraindicated, including patients with renal failure, cross-reactivity to danaparoid or at high hemorrhagic risk. The goal was also to share experience in biological monitoring of elderly intensive care unit (ICU) patients using anti-IIa activity measured by modified thrombin time (TT) and ecarin clotting time (ECT).

## Methods

### Study design and patients

We conducted an open-label, multicenter clinical study between September 10, 2009 and February 2, 2011 in 11 French centers where a total of 57 patients (older than 18 years) were screened if they had suspected HIT and required parenteral anticoagulation. Only patients with a local diagnosis of HIT with or without ongoing thrombosis were finally included. Patients with uncontrolled bleeding or severe hepatic impairment (Child-Pugh Class C) were excluded.

The study was conducted in compliance with the International Conference on Harmonization Good Clinical Practice Guidelines and the Declaration of Helsinki. The protocol was approved by the Independent Ethics Committee and the French competent authority, ANSM (Agence Nationale de Sécurité du Médicament). Each patient provided written informed consent.

### Diagnosis of HIT

Laboratory diagnosis of HIT was based on the positivity of at least one of the following biological tests: an enzyme-linked immunosorbent assay (ELISA), a platelet aggregation test (PAT) or a serotonin release assay (SRA). Clinical diagnosis was established by an independent scientific committee (ISC).

### Argatroban dosing

The recommended starting dose for this study was 1 μg/kg/min. Nevertheless, for patients with moderate hepatic impairment (Child-Pugh Class B) or at risk of decreased hepatic perfusion, such as those requiring intensive care with congestive heart failure, multi-system organ failure or following cardiac surgery, and with hemorrhagic risk, the recommended starting dose was 0.5 μg/kg/min.

Treatment was continued until anticoagulation was no longer required or until adequate oral anticoagulation was achieved. The aPTT value was checked at baseline and 2 hours after starting treatment and the dose adjusted until the steady state aPTT was within the desired therapeutic range (i.e., 1.5–3.0 times the pre-argatroban baseline value as measured 10–60 minutes prior to the start of infusion but not exceeding 100 seconds). Patients were assessed from baseline, during treatment and 24 hours after the end of argatroban infusion, with a follow-up observation at 30 days after the end of the treatment, or hospital discharge.

### Monitoring of laboratory parameters

Laboratory tests were carried out in each participating center using local protocols and methods to support diagnosis and treatment. A centralized laboratory performed clotting time measurements on the frozen plasma samples from all sites on a BCS Coagulation Analyzer (Siemens, Saint-Denis, France) to allow for comparison between patients and methods. Measurement of the aPTT was performed using a PTT automate (Diagnostica Stago, Asnières, France). Anti-IIa activity was determined using ecarin clotting time (ECT) and modified thrombin time (TT). The modified TT was measured using the Hemoclot® thrombin inhibitor assay (Hyphen, Biomed, Neuville, France). The ECT was measured using ecarin, a purified protease, (Diagnostica Stago, Asnières, France) diluted in Hepes 0.025 M CaCl_2_ buffer at a final concentration of 5 IU/ml. All clotting times were expressed in seconds. ECT and TT measurements were then converted into direct concentration units of argatroban (μg/ml) using the same calibration curve (Hyphen, Biomed, Neuville, France).

### Argatroban treatment outcome assessments

The primary endpoint was a composite of all-cause death, thrombosis (new and extended) and amputation. Secondary efficacy endpoints included the individual components of the primary endpoint and death related to HIT and major/minor bleeding. The laboratory efficacy endpoints included time to platelet recovery, aPTT and anti-IIa (onset and maintenance). All adverse events (AEs) were documented, whether considered related or not related in any way to the study drug(s). An independent scientific committee (ISC) including clinicians and biologists reviewed subject enrollment and adjudicated HIT diagnosis and endpoints.

### Statistical analysis

Exploratory statistical data analysis was performed with descriptive interpretation of intra-individual comparisons using simple univariate statistics (mean, standard deviation, median, minimum, maximum, differences to baseline; paired *t* test percentages and confidence intervals if applicable). All tests were two-sided at the 5 % level of significance. In the case of significant departures from the normal distribution as measured by a significant result of the Shapiro-Wilk test the data were evaluated with Wilcoxon's signed-rank test (matched pairs).

## Results

Heparin products had been given for prophylaxis in 12 patients (60 %) and/or therapeutic treatment in 10 patients (50 %).

### HIT diagnosis

Of all 57 screened patients, 20 were included in the study on the basis of a local diagnosis of HIT. Treatment with argatroban was started as soon as the diagnosis of HIT was confirmed. Therefore, an alternative (non argatroban) anticoagulant was given to 15 patients in the interim period prior to diagnostic confirmation, for a median duration of 3 days. Thirteen of these received danaparoid, one received danaparoid, coumadin and clopidogrel, and one subject received lepirudin.

In all 20 patients included, the mean platelet count at heparin initiation was 204 ± 61 G/l and decreased to reach a mean nadir platelet count of 56 ± 24 G/l (minimum 25, maximum 113) after a period of 5 to 21 days. Each laboratory performed either an IgG-specific or a polyspecific (IgG, IgA and/or IgM) ELISA. All 20 patients were tested. Sixteen patients (80 %) had a positive result and four patients (20 %) were negative. Sixteen patients (80 %) were tested for platelet aggregation. Ten patients (50 %) had a positive result and four patients (20 %) were negative. Inconclusive results were reported in two patients (10 %). Only four patients (20 %) were tested for serotonin release assay. A positive result was observed in three patients (15 %) and an inconclusive result reported for one patient. Overall, 14 patients had at least a positive ELISA and one positive functional test. Patients 2 and 20 had only a positive ELISA with high optical density (OD) values, 2.97 and 3.31 respectively. Finally, the diagnosis of HIT was adjudicated by the ISC in 16 patients (80 %). The four patients for whom serological tests could not confirm the diagnosis of HIT were still included in the study based upon the strong clinical suspicion of HIT raised by the local physicians. A summary is provided in Table 1.

**Table 1:** Results of clinical and biological heparin-induced thrombocytopenia (HIT) diagnosis evaluation

Patient	HIT experts opinion	« 4T » score	Polyspecific (IgG, IgA or IgM) ELISA OD/cutoff		Platelet aggregation	Serotonin release assay
1	Confirmed	5	1.72/0.52	Positive	Positive	n.a.
2	Confirmed	5	2.97/0.51	Positive	Negative	n.a.
3	Confirmed	7	2.73/0.51	Positive	Positive	n.a.
4	Confirmed	7	3.09/0.46	Positive	Positive	n.a.
5	Confirmed	4	>2.5/0.5	Positive	Negative	Positive
6	Confirmed	6	2.5/0.5	Positive	n.a.	Positive
7	Not confirmed	5	0.37/0.7	Negative	n.a.	n.a.
8	Not confirmed	5	0.18/0.12	Positive	n.a.	Inconclusive
9	Confirmed	8	1.56/0.4	Positive	n.a.	Positive
10	Confirmed	4	2.77/0.46	Positive	Positive	n.a.
11	Confirmed	7	0.84/0.38	Positive	Inconclusive	n.a.
12	Confirmed	6	1.89/0.47	Positive	Positive	n.a.
13	Confirmed	6	0.17/0.48	Negative	Positive	n.a.
14	Confirmed	5	0.93/0.43	Positive	Positive	n.a.
15	Confirmed	5	2.68/0.45	Positive	Positive	n.a.
16	Not confirmed	5	0.06/0.43	Negative	Negative	n.a.
17	Confirmed	5	2.41/0.46	Positive	Positive	n.a.
18	Not confirmed	6	0.08/0.43	Negative	Negative	n.a.
19	Confirmed	6	3.12/0.61	Positive	Positive	n.a.
20	Confirmed	6	3.31/0.45	Positive	Inconclusive	n.a.

*Od* optical density), *n.a.* not applicable, *4T* Warkentin “4T” pre-test probability score [9]

### Demographic and baseline characteristics

The population of the study included twenty patients (14 male and 6 female) with a mean age of 72 ± 10 years (range 41–85, median 73). The overall mean weight and body mass index (BMI) were 78.4 kg (range 55.0–130.0, median 74.5) and 28.1 ± 4.8 kg/m^2^ (range 21.5–40.2, median 27.5) respectively. The majority of patients were in intensive care units (70 %) during the study and 65 % of patients had moderate hepatic impairment (Child-Pugh class B). Renal failure was observed in 14 patients (70 %).

Bleeding and thrombotic risk factors were identified for each patient. The majority of them combined multiple risk factors as expected in critically ill patients (Table 2). Seventy percent of the study patients had thrombosis at enrollment.

**Table 2:** Summary of clinical efficacy outcomes by the Steering Committee

Patient number	Bleeding risk factors	Thrombotic risk factors	HIT	Baseline thrombosis^a^	New/extended thrombosis	Major bleeding	Bleeding	Death
1	Gastric ulcer; renal failure	Recent orthopedic surgery; obesity; vena cava filter	Confirmed	Yes x 2	No	No	No	No
2	Antiplatelet treatment	-	Confirmed	No	No	No	No	No
3	Cardiac surgery; renal failure	AF	Confirmed	Yes	No	Yes, hematuria	No	Yes, cardiogenic shock
4	Antiplatelet treatment	Obesity; DVT; PE; lupus anticoagulant	Confirmed	Yes x 2	Yes x 4	No	No	No
					3 DVT extension			
					1 new			
5	Antiplatelet treatment; renal failure	Sepsis; AF	Confirmed	No	No	No	No	No
6	Acute renal failure; moderate hepatic cystosis	DVT; sepsis	Confirmed	Yes	No	No	No	No
7	Renal failure; cardiac insufficiency; liver insufficiency	DVT; PE	Not confirmed	Yes	No	No	No	Yes, congestive heart failure
8^b^	Acute renal failure; hepatic insufficiency; gastric ulcer; abdominal surgery; overdose	Cancer; sepsis; PE	Not confirmed	Yes x 2	Yes x 2	No	Yes	Yes, MOF but after end of FU period
					2 new			
9	Digestive angiodysplasia; renal failure	AF; cardiac insufficiency	Confirmed	Yes	Yes, new-limb ischemia	No	No	No
10	-	Sepsis; DVT; PE	Confirmed	Yes x 2	No	No	No	Yes, refractory hypoxemia due to ARDS
11	Abdominal and cardiac surgery	Cancer; cardiac surgery; sepsis	Confirmed	Yes x 2	No	No	No	No
12	Hepatic impairment and cardiac surgery	Cardiac surgery	Confirmed	No	No	No	No	No
13	Acute kidney failure; intra-alveolar hemorrhage	Obesity; cardiac insufficiency; sepsis	Confirmed	Yes	No	No	No	No
14^c^	Renal failure; hepatic insufficiency; cardiac surgery	Cardiac insufficiency; intra-aortic balloon pump; cardiac surgery	Confirmed	Yes x 2	No	Yes, rectorrhagia	Yes, hematemesis	Yes, MOF and refractory shock
15	-	Arterial thrombosis; sepsis	Confirmed	Yes	Yes, new right internal jugular vein thrombosis, catheter-related	No	No	No
16	Renal failure; acute hepatic insufficiency	Cardiac insufficiency; AF; varicose vein	Not confirmed	No	No	No	No	No
17	Acute renal failure; vascular surgery	Obesity; AF; sepsis; arterial thrombosis	Confirmed	Yes	Yes	Yes, due to puncture of dialysis catheter	No	Yes, sepsis with MOF
18	Renal insufficiency; fissuring aneurysm	Suspected cancer; vascular surgery; arterial thrombosis; PE	Not confirmed	Yes x 2	No	No	No	Yes, degradation of general condition complicated by per-renal azotemia
19^d^	Renal insufficiency; aortic dissection surgery; antiplatelet therapy	Obesity; cardiac insufficiency; sepsis	Confirmed	No	No	No	No	No
20	Renal failure and cardiac surgery	Obesity; cardiac surgery; cardiac insufficiency	Confirmed	No	No	No	No	No
Total				14 yes	5 yes	3 x major	2 x non major	7 yes (1 died after end of FU period); all deaths due to underlying diseases

^a^Baseline thrombosis: all confirmed thromboses that were ongoing at the start of argatroban infusion. ^b^Patient 8 had an overdose/medication error. The patient was infused 10.5 μg/kg/min for approximately 1 hour as the study nurse mixed up two pumps. No major bleeding occurred. The death of this patient was not included in this study as it occurred outside the study period. ^c^Patient 14 had Child Pugh class C at the time of inclusion.

^d^Patient 19 was treated with danaparoid for 16 days before the start of the argatroban infusion. This patient was no longer in the acute HIT phase

*AF* atrial fibrillation, *ARDS* acute respiratory distress syndrome, *DVT* deep vein thrombosis, *FU* follow up; *PE* pulmonary embolism, *MOF* multiple organ failure

### Argatroban therapy

The initial mean argatroban dose (± SD) was 0.77 ± 0.45 μg/kg/min (median 0.50, maximum 2.00 μg/kg/min), the mean dose during treatment was 0.75 ± 0.4 μg/kg/min and at the end of infusion the mean dose was 0.8 ± 0.60 μg/kg/min (median 0.54, maximum 2.15 μg/kg/min). Thirteen patients had an initial dose ≤0.5 μg/kg/min and seven patients had an initial dose ≥0.97 μg/kg/min. The mean duration of argatroban treatment was 8.5 ± 6.1 days (median 7.5 days).

### Laboratory efficacy outcomes

The mean platelet count ± SD at baseline was 95 ± 55 G/l, which increased to 147 ± 89 G/l at day 3 and to 215 ± 102 G/l 24 hours after the end of the infusion (EOI). There was a statistically significant treatment effect on platelet count at EOI + 24 hours (baseline vs. EOI + 24 hours, *p* <0.001; paired *t* test and Wilcoxon signed rank test). The platelet count recovered (recovery defined as 50 % increase) in 12 of the 17 evaluable patients (60 %) at day 3, and in 14 patients out of 16 evaluable patients by EOI + 24 hours, with 10 of these patients reaching a level ≥200 G/l.

The degree of argatroban anticoagulation was monitored in the different local laboratories using aPTT and anti-IIa activity (TT and ECT). As variability of measurements between local laboratories may have a relevant impact on the comparability of the results, all clotting time measurements were centralized. The overall baseline mean aPTT value increased from 44.8 ± 9.8 sec before the start of infusion to a mean value of 78.1 ± 35.8 sec (ratio 1.7) within the first 2 hours after infusion and a mean value of 73.8 ± 20.7 sec (ratio 1.6) during the entire period under argatroban therapy. The mean value of 77.0 ± 31.1 sec at EOI, based on data from 12 patients who had sufficient data at this time point, was similar to the value at the 2-hour time point.

**Fig. 1:**
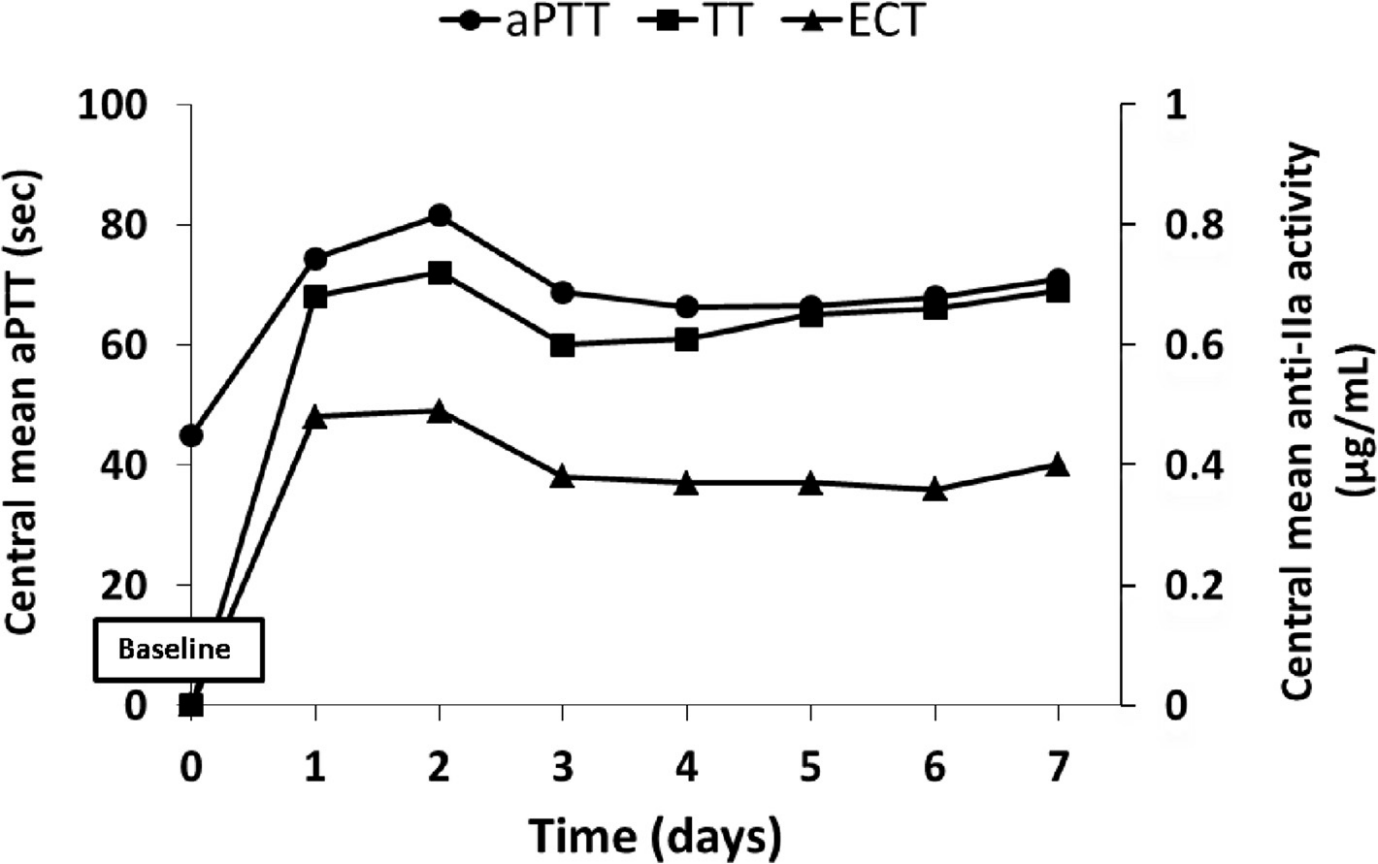
Activated partial thromboplastin time (*aPTT*) and anti-IIa activity measurements versus time. Each point is the mean of *aPTT* or ecarin clotting time (*ECT*) or thrombin time (*TT*) values for a given time in all study patients

Figure 1 illustrates the overall course of the mean, minimum and maximum aPTT values over time. It shows a rapid increase in mean aPTT time at the 2-hour time point after the start of the infusion and stabilization from day 3 onwards, even if the majority of patients were in intensive care units with frequent variability of aPTT values. Measurement of the anticoagulation status of the patient showed that anti-IIa values increased to 0.27 ± 0.31 using ECT at the 2-hour time point after the start of the infusion, indicating the quick onset of action of the argatroban treatment, and reached a final mean value of about 0.34 ± 0.16 μg/ml at the end of the infusion. As with ECT, argatroban concentration using modified TT increased during the first 2 hours to 0.39 ± 0.29 μg/ml. The concentrations reached a final mean of 0.61 ± 0.28 μg/ml at the end of the infusion.

Following argatroban therapy five patients (25 %) benefited from vitamin K antagonist bridge therapy which was monitored using both international normalized ratio (INR) and aPTT values.

### Outcomes

#### Clinical efficacy and safety outcomes

In the study population during treatment and up to 30 days post argatroban treatment, deaths or amputation, or new and/or extended thrombosis were reported in 10 patients (50 %) (Table 2). Six patients died during the study. All deaths were assessed as being due to the patients’ underlying disease (one patient with cardiogenic shock, one with congestive heart failure, one with refractory hypoxemia due to acute respiratory distress syndrome (ARDS), one with multiple organ failure (MOF) and refractory shock, one with sepsis with MOF and one with degradation of their general condition, complicated by renal azotemia). There were no amputations reported in the 20 patients; in particular none of the 3 patients with ischemic limbs had to undergo an amputation.

Five patients (numbers 4, 8, 9, 15 and 17) (25 %) experienced an episode of new and/or extended thrombosis that was diagnosed up to 30 days after argatroban treatment (Table 2). Four of them presented with HIT and thrombosis before starting argatroban therapy. Two of them had a prolonged baseline aPTT due to the presence of lupus anticoagulant (patient 4) and coagulation disorders (patient 17), respectively. At the time of thrombosis diagnosis patients 4, 8, 9 and 15 presented with an aPTT value 1.4-fold to 2.0-fold greater than the baseline value whereas the aPTT prolongation was more than 2.0-fold greater than the baseline value for patient 17 (Table 3). Regarding the anticoagulant status of these patients, results showed that the mean anti-IIa activity value measured by modified TT ranged between 0.43 and 1.95 μg/ml as compared with 0.61 ± 0.28 μg/ml corresponding to the mean anti-IIa activity reported in the study population throughout the duration of the treatment (Table 4).

**Table 3:** Patients having experienced a thrombotic and/or hemorrhagic episode

Thrombotic complications								
	Thrombotic risk factor	Thrombosis at baseline	aPTT at baseline (sec)	Event during argatroban therapy	aPTT at diagnosis of the event (sec)	Dose of argatroban at diagnosis of thrombosis μg/kg/min	Anti-IIa^b^ (TT) at diagnosis of thrombosis (μg/ml)	Mean anti IIa^b^ (TT) (before diagnosis of thrombosis) (μg/ml)
Patient 4	Obesity, lupus anticoagulant	DVT + PE	51.9	D7: DVT extension; D16: DVT extension (iliac vein) + blue toe syndrome; D29: PE recurrence	Mean aPTT ± SD	1.7	0.64	0.43 ± 0.12
					91.3 ± 6.3			
Patient 8^a^	Cancer; sepsis	PE	37.1	D4: new DTV (femoral vein); D16:thrombosis of the femoral and left external iliac vein + right jugular vein	49.5	10.5 (for 1 hour)	0.13	1.95 ± 2.82
Patient 9	AF; cardiac insufficiency	Peripheral ischemia	36.8	D6: ischemia of the right lower limb	61.6	1.75	1.06	0.87 ± 0.35
Patient 15	Sepsis	Cerebral ischemic lesions	39.9	D14: right internal jugular thrombosis	54.9	1.19	0.47	0.61 ± 0.12
Patient 17	Obesity; AF; sepsis	Peripheral ischemia	48.7	D3: obstruction of the right femoral artery	135.8	0.8	1.44	1.51 ± 0.31
Hemorrhagic complications								
	Bleeding risk factor		aPTT at baseline (sec)	Event during argatroban therapy	aPTT at diagnosis of the event (sec)	Dose of argatroban before bleeding μg/kg/min	Anti-IIa^b^ (TT) before bleeding (μg/ml)	Mean anti-IIa^b^ (TT) before bleeding (μg/ml)
Patient 3	Cardiac surgery; renal failure		54.7	Major bleeding at D21: hematuria	63.9	0.62	0.61	0.43 ± 0.13
Patient 8	Acute renal failure; hepatic insufficiency; gastric ulcer; abdominal surgery; overdose		37.1	Minor bleeding after 2-hour infusion: gums and nose	149.7	Medication error	6.15	n.a.
Patient 14	Renal failure; hepatic insufficiency; cardiac surgery		63.9	Minor bleeding at D6: hematemesis; major bleeding at D9: rectorrhagia	66.6	0.12	0.66	0.67 ± 0.29
Patient 17	Acute renal failure; vascular surgery		48.7	Major bleeding at D5: puncture of a dialysis catheter	133	0.32	1.53	1.52 ± 0.22

^a^Infusion was stopped due to medication error. ^b^Anti-IIa activity was expressed as argatroban concentration. Patients 4, 8, 9, 15 and 17 experienced an episode of new or extended thrombosis; patients 3, 8, 14 and 17 experienced bleeding episodes. *aPTT* activated partial thromboplastin time, *ECT* ecarin clotting time, *TT* thrombin time, *AF* atrial fibrillation, *DVT* deep vein thrombosis, *PE* pulmonary embolism, *D* day, n.a. not applicable

**Table 4:** Anti-IIa activity^a^

	Time	Concentration of argatroban mean ± SD (μg/ml)
ECT	2 hours after start	0.27 ± 0.31
	EOI	0.34 ± 0.16
TT	2 hours after start	0.39 ± 0.29
	EOI	0.61 ± 0.28

^a^Anti-IIa activity was measured using ecarin clotting time (*ECT*) and thrombin time (*TT*) and expressed as argatroban concentration. Data were determined by the central laboratory. *EOI* end of infusion

Four patients (numbers 3, 8, 14 and 17) (20 %) reported a total of five bleeding events; three events were adjudicated as major bleeding while the other two events were adjudicated as minor (Table 3). At diagnosis of bleeding, these patients presented with an aPTT value one-fold to four-fold greater than the baseline value. The mean anti-IIa activity values measured by modified TT ranged between 0.43 and 1.52 μg/ml as compared with the mean anti-IIa activity reported in the study population, 0.61 ± 0.28 μg/ml.

### Adverse events

Amongst the reported AEs, 21 were considered to be either possibly or probably related to argatroban treatment (adverse drug reactions, ADRs) and they occurred in nine patients who exhibited at least one thrombotic or hemorrhagic factor (Table 2). The majority of these ADRs were related to thrombotic and hemorrhagic events and included pulmonary embolism, blue toe syndrome, femoral artery embolism, jugular vein thrombosis, pelvic vein thrombosis, rectal hemorrhage, puncture site hemorrhage, and hematuria.

## Discussion

HIT is considered a clinico-pathological syndrome for which the diagnosis is based on thrombocytopenia with or without thrombosis and the detection of heparin-dependent antibodies [9]. Its recognition and diagnosis remain challenging in the ICU setting [10–12]. In the present study in which most of patients were in ICU, the diagnosis of HIT was validated *a posteriori* in 16 out of 20 patients after adjudication by an ISC. The diagnosis was based on a series of commonly used clinico-biological criteria and supported by requirement for at least one positive serological test. We note that serological confirmation of a diagnosis of HIT has been rarely reported in previously published studies describing outcomes of argatroban-treated patients.

Because most of the study patients were at high hemorrhagic risk the initial mean dose of argatroban was low (0.77 ± 0.45 μg/kg/min). The recommended initial dosage of argatroban in adult patients without hepatic impairment is 2 μg/kg/min [13]. However, based on clinical experience gained from past studies and from marketed drugs, dosing schedules had to be optimized according to specific subjects’ condition, particularly in the case of ICU settings. To date there are limited published data on dosing patterns for argatroban therapy in the ICU. Maintenance doses of 0.125–0.91 μg/kg/min have been reported in case reports and small studies of 4 to 65 patients [14, 15]. Furthermore, Saugel et al. showed that argatroban should be initiated at a markedly reduced dose of about one tenth to one eighth of the recommended dose in ICU patients with multiple organ dysfunction syndrome [16]. Moreover, initial infusions of 1.5–2 μg/kg/min in these patients have resulted in supratherapeutic aPTTs and bleeding [14, 15]. One group of authors suggested that passive hepatic congestion, accumulation of metabolites, and/or interactions with multiple medications used in the ICU may justify lower dose requirements [17]. In the present study, where most of the patients were treated in an ICU, low argatroban doses were sufficient to achieve the target aPTT values, and were associated with a rapid reversal of thrombocytopenia in the majority of patients. This initial rate was in accordance with the one used in the study of Link et al. (continuous infusion of 0.7 μg/kg/min), who investigated argatroban treatment in 30 critically ill patients with HIT receiving continuous renal replacement therapy [18]. In contrast, this dose was higher than the argatroban dose applied in the study of Saugel et al. (0.24 μg/kg/min) performed in 12 elderly patients with multiple organ dysfunction, probably reflecting the degree of illness severity in these patients [16].

While under argatroban therapy we observed a rapid reversal of thrombocytopenia in the majority of patients. During argatroban treatment the aPTT assay is commonly used for therapeutic monitoring. In critically ill patients, coagulation abnormalities are commonly found and may prolong the baseline aPTT [19]. In the present study the baseline aPTT was already prolonged in 13 patients. Importantly, abnormalities in the coagulation tests associated with HIT may confound its management when only a global coagulation assay is used to monitor anticoagulation. Warkentin et al. raise the importance of baseline elevation of aPTT prior to initiating anticoagulant therapy in the risk of subsequent anticoagulation failure due to aPTT confounding, which is misleading with respect to indicating a patient’s true level of anticoagulation [20]. In this context alternative methods allowing for measurement of actual concentration in patient plasma are of interest [21]. Anti-IIa activity has been reported as a more specific monitoring parameter for direct thrombin inhibitors (DTIs), in particular in the ICU setting where close monitoring is needed [22]. The ECT and TT assays activate the clotting cascade at the level of thrombin generation and neither of these tests contains phospholipid. Consequently neither is affected by variation of most of the coagulation factors or by the presence of lupus inhibitors [23, 24]. It is noteworthy that the present study showed that the two methods were not superimposable with higher values with modified a TT compared with the ECT (ratio TT/ECT of about 1.5).

Nevertheless, a common limitation in monitoring any DTI with anti-IIa activity is the lack of well-defined therapeutic concentration ranges. Indeed, the therapeutic ranges are generally based on prolongation of the aPTT and not on drug concentration levels [21]. We extrapolated a theoretical therapeutic range of argatroban from an aPTT standard curve, which was established using a control plasma pool spiked with different concentrations of argatroban, in the range of 0–3 μg/ml. The aPTT of each argatroban concentration was measured and the results (aPTT (sec)/argatroban concentration (μg/ml) were plotted (data not shown); the range for the aPTT of 1.5–3.0 times the baseline value corresponds to a range of 0.25–1.5 μg/ml of argatroban. In our study thrombotic complications occurred in five patients (25 %). Interestingly, these five patients had at least one thrombosis before argatroban initiation. This observation suggests that patients with an initial thromboembolic event at HIT diagnosis were more likely to present a future thromboembolic event as compared with only isolated thrombocytopenia, as already suggested [25] and should probably receive higher doses of argatroban. At the same time, dose limitation should be driven by the presence of high hemorrhagic risk in these patients. In our study the incidence rate of thrombotic complications under argatroban therapy is in agreement with that previously reported. Lewis et al. reported a prospective, historical-controlled study evaluating the efficacy and safety of argatroban as anticoagulant therapy; in patients with isolated HIT a new thrombosis occurred in 8.1 % of argatroban-treated patients versus 22.4 % in control patients with HIT, but in patients with HIT and thrombosis (HITT) the incidence was 19.4 % in the argatroban group versus 34.8 % in control patients with HIT [26]. Our results are in agreement with this study, with a higher incidence of new thrombosis in HIT patients with initial thrombosis. Sakr et al. reported thrombotic complications in 35.7 % of patients from a cohort of 88 surgical ICU patients with positive HIT antibodies treated with danaparoid or argatroban [27]. Considering that the initial mean dose of argatroban administered to our patients was low due to both high hemorrhagic and thrombotic risks, our study did not report more new thromboses. Looking closely at patients who experienced a new thrombosis, two patients, numbers 4 and 17, presented with a prolonged aPTT at baseline, due to the presence of lupus anticoagulant (patient 4) and probably related to DIC (patient 17). Patient 4 experienced four episodes of thrombosis and was probably undertreated. In patients with HIT and lupus anticoagulant who do not respond to initial doses of infusion and may need the target aPTT increased, higher doses of argatroban should be considered [28] and the monitoring should use anti-IIa activity rather than aPTT. Patient 17 experienced one new thrombotic episode. A baseline elevated aPTT related to DIC can rise to much higher values under DTI therapy, potentially suggesting, falsely, that the patient is over-anticoagulated and making the monitoring particularly difficult. It is therefore of interest to use anti-IIa activity as well as aPTT. The anti-IIa values before diagnosis of thrombosis and at diagnosis of thrombosis were high, suggesting that the thrombotic event was not related to drug under-dosing. Moreover, considering all study patients who experienced a new episode of thrombosis and based on the theoretical therapeutic range of argatroban concentrations mentioned above, only patient 4 was at the lower limit of this window.

For other anticoagulants, Elalamy et al. reported 19 % thrombotic complications (arterial or venous) in 114 HIT patients treated with danaparoid, in a multicenter, retrospective, case-control study performed in seven French University Hospital centers [25]. In a retrospective observational analysis in 181 patients with confirmed HIT and treated with lepirudin, Tardy et al. reported that 13.8 % of patients experienced a thrombotic event [29].

A total of five bleeding events occurred in four patients, of which three events were adjudicated as major bleeding (15 %). In previous studies such events affected 5–20 % of patients with HIT, depending on the treatment drug and population studied [25, 27, 29]. Elalamy et al. found an overall rate of major bleeding of 9 % in danaparoid-treated patients and Tardy et al. reported 20.4 % of major bleeding events in lepirudin-treated patients [25, 29]. Overall, although indirect comparisons between studies should be interpreted with caution, it appears that the incidence of new thrombosis is higher with argatroban compared with lepirudin and danaparoid. However, bleeding events tend to be lower compared with lepirudin.

### Study limitations

Regarding the limitations of this study, it has to be noted that the sample was small. There were also certain constraints in that patients were generally not treated until confirmation of diagnosis, which meant that other anticoagulants such as danaparoid or lepirudin had to be used after the onset of HIT, but before confirmative test results were available and thus, before argatroban initiation. As a result, in 15 patients there was a delay between confirmed diagnosis of HIT and treatment, outside the 48-hour window. Adjudication by the ISC led to recommendations later in the course of the study, to start treatment before diagnosis was confirmed for patients in whom earlier treatment might have been more beneficial.

## Conclusion

As expected from its pharmacologic properties, argatroban was well-tolerated in patients with renal impairment who were mostly in the ICU. The overall risk–benefit assessment of the ISC members was favorable for argatroban. In conclusion, it can be stated that considering its hepatic elimination, its short half-life and the low rate of hemorrhagic complications in the study patients, argatroban can be considered as a safe therapeutic option in HIT patients at high hemorrhagic risk and with renal failure, particularly in an ICU setting.

## Key messages

- Argatroban can be considered as a safe therapeutic option in elderly patients with HIT and at high hemorrhagic risk, particularly in an ICU setting
- In critically ill patients, the dosing of argatroban cannot be only adjusted with a global anticoagulation assay, aPTT
- The use of aPTT in addition to anti-IIa measurement is essential for argatroban monitoring
- The two methods, modified TT and ECT, used to measure anti-IIa activity, are not superimposable with higher values with TT compared with ECT

## Abbreviations

ADR: adverse drug reaction
AE: adverse event
aPTT: activated partial thromboplastin time
ARDS: acute respiratory distress syndrome
BMI: body mass index
DIC: dissiminated intravascular coagulation
DTI: direct thrombin inhibitor
DVT: deep vein thrombosis
ECT: ecarin clotting time
ELISA: enzyme-linked immunosorbent assay
EOI: end of infusion
FAS: full analysis set
HIT: heparin-induced thrombocytopenia
ICU: intensive care unit
INR: international normalized ratio
ISC: independent scientific committee
MOF: multiple organ failure
PAT: platelet aggregation
PE: pulmonary embolism
PP: per-protocol
SAF: safety population
SRA: serotonin release assay
TEAE: treatment-emergent adverse event
TT: thrombin time
VKA: vitamin K antagonist

## References

[1] Alatri A, Armstrong AE, Greinacher A, Koster A, Kozek-Langenecker SA, Lance MD (2012). Results of a consensus meeting on the use of argatroban in patients with heparin-induced thrombocytopenia requiring antithrombotic therapy - a European Perspective. Thromb Res..

[2] Tang IY, Cox DS, Patel K, Reddy BV, Nahlik L, Trevino S (2005). Argatroban and renal replacement therapy in patients with heparin-induced thrombocytopenia. Ann Pharmacother..

[3] Magnani HN, Gallus A (2006). Heparin-induced thrombocytopenia (HIT). A report of 1,478 clinical outcomes of patients treated with danaparoid (Orgaran) from 1982 to mid-2004. Thromb Haemost.

[4] Hursting MJ, Murray PT (2008). Argatroban anticoagulation in renal dysfunction: a literature analysis. Nephron Clin Pract..

[5] Walenga JM, Ahmad S, Hoppensteadt D, Iqbal O, Hursting MJ, Lewis BE (2002). Argatroban therapy does not generate antibodies that alter its anticoagulant activity in patients with heparin-induced thrombocytopenia. Thromb Res..

[6] Linkins LA, Dans AL, Moores LK, Bona R, Davidson BL, Schulman S (2012). Treatment and prevention of heparin-induced thrombocytopenia: Antithrombotic Therapy and Prevention of Thrombosis, 9th ed: American College of Chest Physicians Evidence-Based Clinical Practice Guidelines. Chest..

[7] Lassila R, Antovic JP, Armstrong E, Baghaei F, Dalsgaard-Nielsen J, Hillarp A (2011). Practical viewpoints on the diagnosis and management of heparin-induced thrombocytopenia. Semin Thromb Hemost..

[8] Periodic Safety Update Report for Argatroban. In: Mitsubishi Tanabe Pharma Corporation; 1 February 2011 - 31 July 2011.

[9] Warkentin TE (2003). Heparin-induced thrombocytopenia: pathogenesis and management. Br J Haematol..

[10] Bates D, Griffin S, Angel B (2009). Clinical experience with argatroban for heparin-induced thrombocytopenia in a large teaching hospital. Can J Hosp Pharm..

[11] Greinacher A, Selleng K (2010). Thrombocytopenia in the intensive care unit patient. Hematology Am Soc Hematol Educ Program..

[12] Galvagno SM, Hong CM, Lissauer ME, Baker AK, Murthi SB, Herr DL (2013). Practical considerations for the dosing and adjustment of continuous renal replacement therapy in the intensive care unit. J Crit Care..

[13] Transparency Committee on Arganova®. In: The Haute Autorité de santé (HAS) - or French National Authority for Health 16 November 2011.

[14] Williamson DR, Boulanger I, Tardif M, Albert M, Gregoire G (2004). Argatroban dosing in intensive care patients with acute renal failure and liver dysfunction. Pharmacotherapy..

[15] Beiderlinden M, Treschan TA, Gorlinger K, Peters J (2007). Argatroban anticoagulation in critically ill patients. Ann Pharmacother..

[16] Saugel B, Phillip V, Moessmer G, Schmid RM, Huber W (2010). Argatroban therapy for heparin-induced thrombocytopenia in ICU patients with multiple organ dysfunction syndrome: a retrospective study. Crit Care..

[17] Begelman SM, Baghdasarian SB, Singh IM, Militello MA, Hursting MJ, Bartholomew JR (2008). Argatroban anticoagulation in intensive care patients: effects of heart failure and multiple organ system failure. J Intensive Care Med..

[18] Link A, Girndt M, Selejan S, Mathes A, Bohm M, Rensing H (2009). Argatroban for anticoagulation in continuous renal replacement therapy. Crit Care Med..

[19] Levi M, Opal SM (2006). Coagulation abnormalities in critically ill patients. Crit Care..

[20] Warkentin TE (2014). Anticoagulant failure in coagulopathic patients: PTT confounding and other pitfalls. Expert Opin Drug Saf..

[21] Love JE, Ferrell C, Chandler WL (2007). Monitoring direct thrombin inhibitors with a plasma diluted thrombin time. Thromb Haemost..

[22] Harder S, Graff J, Klinkhardt U, von Hentig N, Walenga JM, Watanabe H (2004). Transition from argatroban to oral anticoagulation with phenprocoumon or acenocoumarol: effects on prothrombin time, activated partial thromboplastin time, and Ecarin Clotting Time. Thromb Haemost..

[23] Pendleton R, Wheeler MM, Rodgers GM (2006). Argatroban dosing of patients with heparin-induced thrombocytopenia and an elevated aPTT due to antiphospholipid antibody syndrome. Ann Pharmacother..

[24] Nowak G (2003). The ecarin clotting time, a universal method to quantify direct thrombin inhibitors. Pathophysiol Haemost Thromb..

[25] Elalamy I, Tardy-Poncet B, Mulot A, de Maistre E, Pouplard C, Nguyen P (2009). Risk factors for unfavorable clinical outcome in patients with documented heparin-induced thrombocytopenia. Thromb Res..

[26] Lewis BE, Wallis DE, Berkowitz SD, Matthai WH, Fareed J, Walenga JM (2001). Argatroban anticoagulant therapy in patients with heparin-induced thrombocytopenia. Circulation..

[27] Sakr Y, Haetscher F, Gonsalves MD, Hoffman M, Theis B, Barz D (2012). Heparin-induced thrombocytopenia type II in a surgical intensive care unit. J Crit Care..

[28] Hellwig TR, Peitz GJ, Gulseth MP (2012). High-dose argatroban for treatment of heparin-induced thrombocytopenia with thrombosis: a case report and review of laboratory considerations. Am J Health Syst Pharm..

[29] Tardy B, Lecompte T, Boelhen F, Tardy-Poncet B, Elalamy I, Morange P (2006). Predictive factors for thrombosis and major bleeding in an observational study in 181 patients with heparin-induced thrombocytopenia treated with lepirudin. Blood..

